# Polysaccharides from natural resource: ameliorate type 2 diabetes mellitus via regulation of oxidative stress network

**DOI:** 10.3389/fphar.2023.1184572

**Published:** 2023-07-11

**Authors:** Li-Ying He, Yong Li, Shu-Qi Niu, Jing Bai, Si-Jing Liu, Jin-Lin Guo

**Affiliations:** ^1^ Key Laboratory of Characteristic Chinese Medicine Resources in Southwest China, College of Pharmacy, Chengdu University of Traditional Chinese Medicine, Chengdu, China; ^2^ College of Medical Technology, Chengdu University of Traditional Chinese Medicine, Chengdu, China; ^3^ Chongqing Key Laboratory of Sichuan-Chongqing Co Construction for Diagnosis and Treatment of Infectious Diseases Integrated Traditional Chinese and Western Medicine, Chongqing, China

**Keywords:** diabetes mellitus, natural polysaccharide, oxidative stress, metabolism, mechanism

## Abstract

Diabetes mellitus (DM) is a group of metabolic diseases characterized by hyperglycemia that can occur in children, adults, elderly people, and pregnant women. Oxidative stress is a significant adverse factor in the pathogenesis of DM, especially type 2 diabetes mellitus (T2DM), and metabolic syndrome. Natural polysaccharides are macromolecular compounds widely distributed in nature. Some polysaccharides derived from edible plants and microorganisms were reported as early as 10 years ago. However, the structural characterization of polysaccharides and their therapeutic mechanisms in diabetes are relatively shallow, limiting the application of polysaccharides. With further research, more natural polysaccharides have been reported to have antioxidant activity and therapeutic effects in diabetes, including plant polysaccharides, microbial polysaccharides, and polysaccharides from marine organisms and animals. Therefore, this paper summarizes the natural polysaccharides that have therapeutic potential for diabetes in the past 5 years, elucidating their pharmacological mechanisms and identified primary structures. It is expected to provide some reference for the application of polysaccharides, and provide a valuable resource for the development of new diabetic drugs.

## 1 Introduction

Diabetes mellitus (DM) is a metabolic disease with symptoms of hyperglycemia. It can be caused by the deficiency of insulin secretion or insulin action in islet β cells (Galicia-Garcia et al., 2020). In 2015, approximately 415 million people worldwide had diabetes; of them, more than 90% had type 2 DM (T2DM). This number is projected to increase to 642 million in 2040 (Chen, 2015; Chatterjee et al., 2017). In addition, the onset of T2DM is often associated with conditions such as chronic hyperglycemia, hyperlipidemia and hypertension, placing a great burden on the healthcare system (Gerich, 2003; Taylor, 2013; Chatterjee et al., 2017). Physical activity has been reported to improve symptoms in diabetic patients with OS (Leeuwenburgh et al., 1994; Venkatasamy et al., 2013), but people seem to lack the time to exercise. Nowadays, unhealthy lifestyles and dietary factors are aggravating this condition (Dos Santos et al., 2019; Salazar-Garcia and Corona, 2021). Diabetes is still a key topic worthy of continued attention of scholars.

Insulin resistance is a major feature in T2DM development (Gerich, 2003) and may be caused by an insulin signalling defect, inflammatory cytokines (Halim and Halim, 2019), lipotoxicity (Kelly et al., 2019; Poznyak et al., 2020), glucose transporter defect, amyloid formation for β-cell dysfunction (Eizirik et al., 2020), OS (Dos Santos et al., 2019), mitochondrial dysfunction, excess fatty acid, or lack of the cretin effect (Galicia-Garcia et al., 2020). At present, according to the manifestations of diabetes, many treatment methods are available for T2DM. In addition to injectable preparations such as insulin and insulin analogs, glucagon-like peptide 1and oral hypoglycemic drugs, such as peptidyl peptidase-4 (DPP-4) inhibitors and sodium-glucose co-transporter 2 (SGLT2) inhibitor, are commonly used to treat diabetes (Chatterjee et al., 2017). New methods have recently been used to treat diabetes, including islet transplantation, gene therapy (Green et al., 2018), and combination therapy. Islet transplantation can effectively control blood sugar and prevent long-term complications by increasing the number of islet cells in patients, to reduce their dependence on exogenous insulin and rebuild their physiological regulation of blood sugar (Bertuzzi et al., 2018; Rickels and Robertson, 2019). However, reducing inflammation and adverse effects around transplantation is a problem associated with this method that needs to be urgently resolved. Stem cell transplantation can help increase β cells content. However, the medical evidence of the efficacy and safety of stem cell therapy to support its routine use for T2DM is insufficient (Chatterjee et al., 2017). Moreover, when single drug therapy is insufficient for controlling T2DM, drug combinations can be used to achieve rapid improvement in blood glucose levels. For example, better outcomes can be achieved with a combination of the DPP-4 inhibitor and metformin or TZD pioglitazone, and insulin and metformin, glyburide, and pioglitazone (Cersosimo et al., 2018). Combination therapy appears to be more effective than single-agent therapy, however, complex multi-agent regimens, higher costs, and reduced patient compliance make it difficult to determine the efficacy of combination therapies (Miccoli et al., 2011). Meanwhile, many adverse reactions associated with these drugs, including hypoglycemia and gastrointestinal problems, can cause extremely painful experiences for patients (Ganesan and Xu, 2019). The development of safer and more economical hypoglycemic drugs has become the need of the hour urgent problem for researchers.

As the advantages of traditional Chinese medicine (TCM) in chronic diseases continue to manifest, several TCMs are used in the treatment of diabetes with mild side effects (Ganesan and Xu, 2019; Zheng et al., 2019). Polysaccharide is a type of bioactive macromolecule present in almost every TCM and food, and plays crucial role in new drug development. It have been found to have antioxidant, antitumor, and immune, regulatory properties and can improve intestinal microorganisms and hypoglycemic effects (Wang et al., 2016; Zheng et al., 2019). Several studies have reported that polysaccharides from natural products may improve diabetes symptoms mainly through regulating the OS (Dos Santos et al., 2019; Zheng et al., 2019), inhibiting α-amylase and α-glucosidase (Qiu et al., 2022), enhancing insulin secretion and improving glucose metabolism in a non-insulin-dependent manner. OS has been recognized as a potential causative factor for diabetes (Maritim et al., 2003; Thakur et al., 2018; Kang and Yang, 2020), and therefore, we tried to summarize natural polysaccharides that can act on T2DM through the OS pathway, hoping to provide a data reference for developing of new therapeutic agents for diabetes.

## 2 Relationship between oxidative stress and diabetes mellitus

**FIGURE 1:**
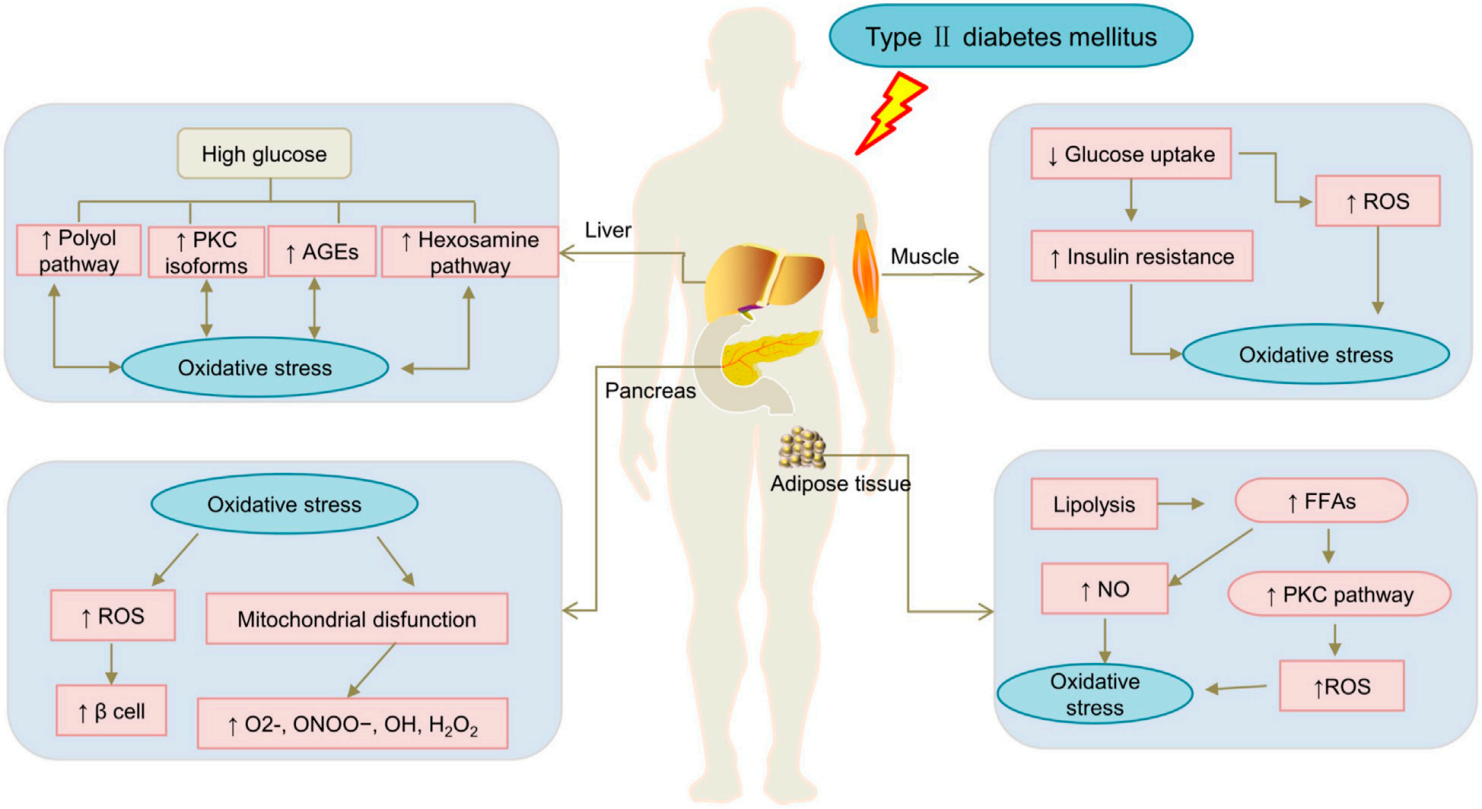
The mechanism of oxidative stress in diabetic patients. AGE, advanced glycosylation end products; FFAs, Free fatty acids. NO, nitric oxide; PKC, protein kinase C; ROS, reactive oxygen species; ↓: downregulated; ↑:upregulated.

Hyperglycemia is a chronic T2DM manifestation, prolonged hyperglycaemia and hyperlipidaemia in T2DM can lead to OS (Nishikawa et al., 2000; Giacco and Brownlee, 2010). OS may aggravate insulin resistance in diabetic patients (Dos Santos et al., 2019). Impaired insulin signalling disrupts the glucose flow into fat cells (Yaribeygi et al., 2019). As the result reactive oxygen species (ROS) and reactive nitrogen species (RNS) overproduction in the cytosol or mitochondria, which counteract the cellular redox balance and induces more OS (Petersen et al., 2003; Ceriello et al., 2009; Salazar-García and Corona, 2021; Liu et al., 2022). Simultaneously, ROS overproduction can damage lipids, proteins, and DNA, leading to the deterioration of β cell function (Omolola et al., 2014; Ezraty et al., 2017). Another momentous reason for OS is the overproduction of free radicals due to mitochondrial dysfunction (Schieber and Chandel, 2014). Furthermore, the levels of plasma free fatty acids (FFAs) are higher in T2DM patients. The increased FFAs levels also affects mitochondrial function, resulting in impaired insulin signalling pathways and lower nitric oxide (NO) levels (Ghosh et al., 2017), and higher ROS contents. Eventually, at high glucose levels, OS induction through regulating the protein kinase C (PKC), glycolysis, hexosamine, polyols pathway, and advanced glycation end products (AGE). In summary, OS plays a pivotal role in the mechanism of DM and related complications (Maritim et al., 2003; Hu et al., 2018). The mechanism of OS in diabetic patients is presented in Figure 1.

### 2.1 Hyperglycemic condition-induced oxidative stress in diabetes

**FIGURE 2:**
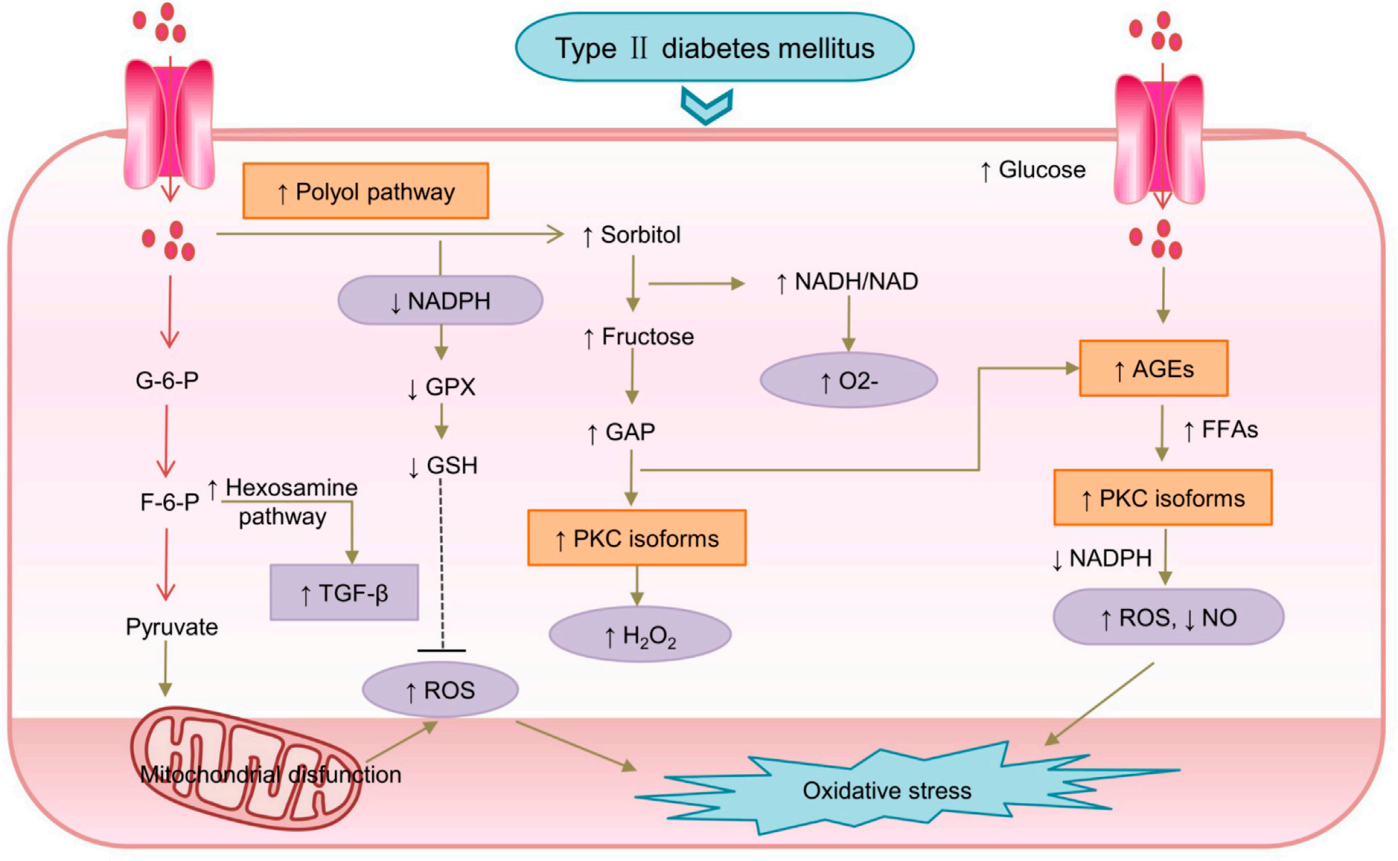
The mechanism of hyperglycemic conditions induced-oxidative stress in diabetes.F-6-P, Fructose-6-P; G-6-P, Glucose-6-P; GSH, glutathione; NO, nitric oxide; TGF-β, transforming growth factor beta; ↓: downregulated; ↑:upregulated.

Constant high glucose levels activate the polyol pathway, and formation of advanced glycosylation end products (AGEs); upregulate the hexosamine pathway (Kang and Yang, 2020); and activate PKC isoforms, leading to OS and eventually worsening of T2DM (Ighodaro, 2018; Thakur et al., 2018). Hyperglycemia influx to the glycolytic pathway and activation of the polyol pathway result in NADH overproduction and higher ROS levels (Mima, 2016). Furthermore, NADPH overproduced leads to a decrease in the intracellular concentration of glutathione (GSH), an antioxidant (Giannini et al., 2011). Moreover, NADPH plays an important role in AGEs-RAGE activation and OS (Ceriello et al., 2009). AGEs are associated with several molecules that enhance oxidative activity, and the activated AGE pathway also ultimately results in increased ROS production (Yamagishi and Matsui, 2010). The higher NADH/NAD^+^ ratio also leads to low-density lipoprotein (LDL) oxidation, cytotoxic effects, decrease in NO levels, and increase of O^2−^ in cells (Eftekharpour and Fernyhough, 2022) (Kessler et al., 1998; Giannini et al., 2011). Excessive production of free radicals damages the body’s antioxidant defense system, and has toxic effects, which cause OS (Halim and Halim, 2019) (Spinelli and Haigis, 2018) (Sergi et al., 2019). The activated polyol pathway also leads to immoderate and continuous activation of several PKC isoforms, which prevents NO release and thus a reduction in NO levels (Goncalves et al., 2022). The mechanism of hyperglycemic condition-induced OS in diabetes is shown in Figure 2.

### 2.2 Mitochondrial dysfunction caused by oxidative stress in diabetes

**FIGURE 3:**
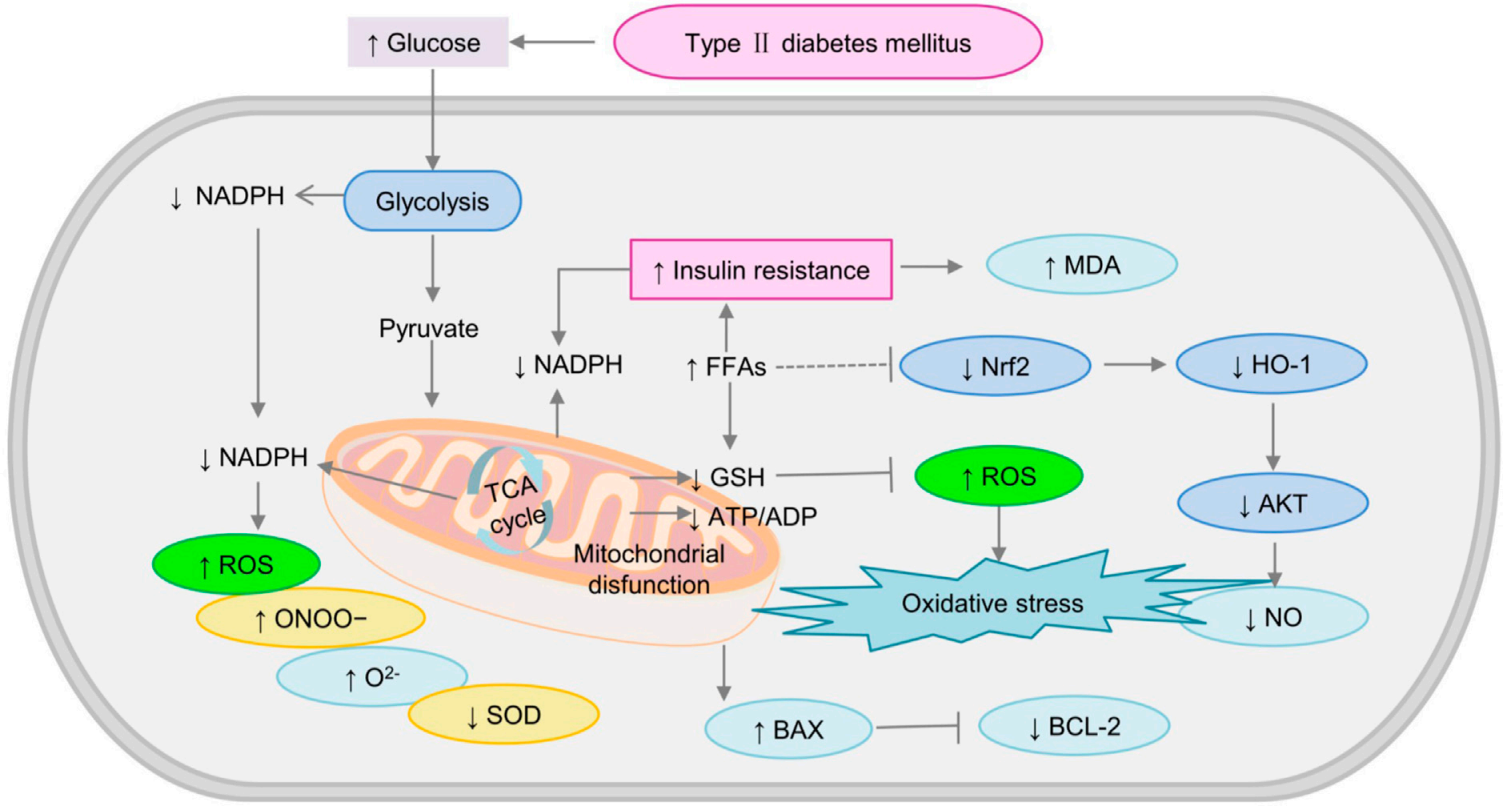
The mechanism of mitochondrial dysfunction induced-oxidative stress in diabetes. AKT, Phosphorylated protein kinase B; BAX, Bcl-2-associated X protein (BAX); HO-1, heme oxygenase-1; MDA, malondialdehyde; Nrf2, nuclear factor erythroid-2; SOD, superoxide dismutase; ↓: downregulated; ↑: upregulated.

Mitochondria, also known as the “energy house” [39], are highly dynamic double-membrane organelles that produce adenosine triphosphate (ATP), the energy that the body requires (Newmeyer and Ferguson-Miller, 2003). Cellular energy production mainly occurs through two pathways: glycolysis and oxidative phosphorylation. Abnormal metabolism in diabetes can lead to excess production of mitochondrial superoxide in endothelial cells (Kaneto et al., 2016), thus damaging the mitochondrial respiratory chain (Maritim et al., 2003; Hu et al., 2018). Mitochondrial dysfunction that occurs in T2DM is inseparable from the glycolytic pathway, presumably it involves increased ROS production and energy expenditure (Qi et al., 2017). It also leads to the production of more free radicals (O^2−^, ONOO^−^, OH, and H_2_O_2_) (Erejuwa, 2012), ROS production (Wu et al., 2018), and ultimately decrease glucose homeostasis and insulin sensitivity in the liver (Yang et al., 2010). In addition to their role in energy production, mitochondria are responsible for FFA synthesis, which is an important consideration in DM (Yao L. et al., 2022). Especially in some obese diabetic patients, FFAs increased significantly (Ghosh et al., 2017). Excessive exposure to FFAs results in mitochondrial dysfunction and the increased formation of the toxic product MDA (Wu et al., 2018). In conclusion, mitochondrial dysfunction accelerates T2DM the development as shown in Figure 3.

## 3 Application of natural polysaccharides in treating diabetes by reducing oxidative stress

As a kind of complex biological macromolecule, polysaccharides have many structural characteristics, such as molecular weight, monosaccharide composition, glucoside bond, branching degree and high chain conformation, which are important factors affecting their functional properties. Due to their near non-toxic properties (Ganesan and Xu, 2019), the activity of natural polysaccharides has received more attention. With the deepening of the research, the chemical structure of polysaccharide and its mechanism of action in the treatment of diabetes have been reported more. It can control diabetes and reduce its complications through various mechanisms. For example, polysaccharides purified from saffron, curcumin, cinnamon and garlic have clear antioxidant properties, and those purified from pumpkin, wolfberry, sea cucumber, mushroom, tea, and beans have good effects on glucose homeostasis, reduce diabetic complications, and ultimately improve insulin sensitivity through the anti-OS damage defense mechanism (Liu et al., 2016). Thus, natural polysaccharides have great potential in improving OS and treating diabetes. In order to accelerate the study of the structure and therapeutic mechanism of natural polysaccharides, we classified the antioxidant polysaccharides with the potential to treat diabetes into 4 categories according to the sources of natural polysaccharides, such as plant polysaccharides, microbial polysaccharides, Marine polysaccharides, and animal polysaccharides.

### 3.1 Polysaccharides derived from plants

#### 3.1.1 Medicinal plant

The *Abelmoschus esculentus* polysaccharides (AEP) were reported to exhibit anti-oxidative and hypoglycemic activities. On the one hand, AEP can significantly increase the content of glutathione peroxidase (GSH-Px), superoxide dismutase (SOD), SOD2, GSH, and catalase (CAT), whereas decreases the content of ROS and malondialdehyde (MDA). In particular, It may alleviate T2DM by improving the phosphoinositide 3-kinase (PI3K)/Phosphorylated protein kinase B (AKT)/glycogen synthase kinase 3 beta (GSK3β) pathway, and activating nuclear factor erythroid-2/heme oxygenase-1(Nrf2/HO-1) pathway. Furthermore, AEP regulated mitochondrial dysfunction by inhibiting NOX2 activation in HFD and STZ treated mice (Liao et al., 2019a). Moreover, AEP obviously increased adenosine monophosphate-activated protein kinase (p-AMPK/AMPK), and exhibited anti-apoptotic effects by diminishing the levels of cleaved caspase-3, and bcl-2-associated X protein (Bax), and enhanced Bcl-2 expression in the same models (Liao et al., 2019b). These indicate that AEP can not only improve the content of antioxidant enzymes, but also improve mitochondrial function and oxidation-related pathways to relieve diabetic.

The *Cyclocarya paliurus* polysaccharide (CCPP) could alleviate T2DM symptoms by boosting GSH-px, SOD, GSH, and CAT levels in diabetic rats (Zhang et al., 2021). In addition, compared with T2DM group, CCPP could upregulate SOD, CAT, GSH-Px, and GSH levels, whereas downregulated AGE and transforming growth factor-beta1 (TGF-β1) levels (Xia et al., 2020; Lin C. et al., 2021). Besides, CCPP significantly improved pathways closely with the nutrition metabolism (amino acids and purine) and energy metabolism (TCA cycle) to improve mitochondrial function (Li et al., 2021). Therefore, CCPP may improve the OS of diabetic patients by regulating AGE pathway to increase the content of antioxidant enzymes. In addition, in the same article, it was also reported to have a role in regulating inflammation and mitochondrial function.

Polysaccharides from the Chinese medicine *Fructus Corni* (FCPs) showed hypoglycemic, hypolipidemic and antioxidative effects in the STZ-induced diabetic rats by increased insulin secretion and promoted pancreatic β cell proliferation. In same study, FCPs have been found to increase the levels of antioxidant enzymes, such as SOD and GSH in STZ injected rats to alleviate diabetic (Wang D. et al., 2019). In a word, the potential mechanism of the hypoglycemic and hypolipidemic activities of FCP in diabetic related to OS. FCPC might have potential applications in regulating OS and T2DM.

The *Astragalus membranaceus* polysaccharide (AMP), as a main bioactive macromolecules of *A*. *membranaceus* (Fisch.), alleviates OS to relieve liver damage in T2DM mice (Chen et al., 2022). One possible therapeutic mechanism was observed in experimental mice that exhibited augmented SOD, GSH, and CAT levels, and decreased MDA levels after AMP treatment. In STZ-treated mice and heterozygous (SOD2^+/−^) knockout mice, astragalus polysaccharides (APS) improved the damage of the cardiac stem and progenitor cells by inhibiting of apoptosis (Chen et al., 2018). In particular, APS has a beneficial effect on SOD2 enzyme activities. Notably, APS reduced cellular ROS levels, and inhibited OS injury indicators, that are 8-Hydroxy-2′-deoxyguanosine (8-OH-dG) and nitrotyrosine levels, in high glucose-induced H9C2 cells (Sun et al., 2019). Moreover, APS can promote SOD, and GSH-Px levels and diminish ROS, MDA, and NO levels in an AGE-induced H9C2 cell model, thereby performing the anti-oxidative function (Chang et al., 2018). Otherwise, APS may display a protective function by activating NGR1/ErbB signalling and the downstream AKT/PI3K signalling pathway (Chang et al., 2018). APS can regulate multiple pathways to improve the etiology and complications of DM (Zhang Z. et al., 2019).

Polysaccharides from *Hedysarum alpinum* (HPS) are an active ingredient for DM treatment. HPS markedly revises OS damage in DM mouse models and cell model. Mechanistically, it inhibits Keap1 signalling and upregulates Nrf2 signalling pathway. Besides, HPS significantly suppressed MDA concentration and enhanced the concentration of antioxidant enzymes in HG-induced Schwann cells. Moreover, HPS stimulated Nrf2 signalling, whereas decreased Keap1 expression in SCs (He et al., 2022). Therefore, HPS has been demonstrated to have antioxidant activity both *in vivo* and *in vitro* and can be considered as a potential diabetes treatment.

The *Morus alba* polysaccharide (MLP) can ameliorate diabetes by improving OS injury and the mitochondrial function of islet cells in HFD- and STZ-induced diabetic rats. Furthermore, MLP inhibits MDA production, but promotes SOD and SDH activities (Liu et al., 2017). In 2015, MLP was found to activate the PI3K/AKT pathway and mitigate OS in HFD- and STZ-induced rats (Ren et al., 2015). These results suggest that MLP has the potential to treat diabetes. MLP could improve OS by reducing MDA content, enhancing SOD, CAT, and GPx activities, and regulating amino acid and lipid metabolism by decreasing TG and TC levels. Additionally, blood urea nitrogen (BUN), albumin (ALB), Creatinine (CRE), and UA (uric acid) levels, and the expression of PI3K of insulin receptor substrate 2 (IRS2) and the PKB/AKT pathway relative to insulin signalling were significantly increased with the MLPs. Finally, glucose levels were restored in T2DM rats (Li et al., 2022).

The *Dendrobium officinale* polysaccharide (DOP) can ameliorate hyperglycemia, improve β-cell function inflammation, regulate lipid concentration, inhibit insulin resistance, and regulate OS to ameliorate diabetes (Yang J. et al., 2020). Specifically, compared with normal C57BL/6 male mice, SOD and CAT concentrations in the liver were significantly increased after DOP administration in T2DM rats (Yang J. et al., 2020). DOP can also improve AKT signalling pathways (Liu et al., 2020b).

*Polygonatum sibiricum* polysaccharides (PSP), as an antioxidant activity component, have been found to have anti-diabetic effects. PSP attenuates diabetes by decreasing ROS production and MDA levels, and augmenting SOD and GPx activities. PSP also scavenged the activation of the Nrf2/HO-1 pathway in HG-induced ARPE-19 cells. Moreover, PSP significantly regulated apoptosis by diminishing bax and caspase-3 expression, and promoting Bcl-2 production (Wang et al., 2022). In another *in vivo* study, PSP was advantageous in controlling polydipsia, polyuria, polyphagia, and weight loss in DM rats. With PSP, MDA content was lowered, and cataract progression was delayed. Thus, PSP may alleviate the progression of diabetes by alleviating OS (Wang et al., 2017d). Simultaneously, PSP pre-treatment significantly alleviated the IR effect in IR-3T3-L1 adipocytes by inhibiting IL-6, IL-1β, and TNF-α levels and promoting Nrf2 and HO-1 expression (Cai et al., 2019).

The polysaccharide (CLP) of *Codonopsis lanceolata*, as an important bio-active, has been used to treat diabetes. It improves insulin sensitivity by regulating Akt pathway and hyperphosphorylation of IRS-1, decrease MDA levels, elevates SOD, and CAT levels, and the GSH/oxidized glutathione ratio, and activates Nrf2 signalling in chronic high fat/high sucrose (HFHS) diet-fed mice (Zhang et al., 2020). CLP through activating Nrf2 signaling pathway and increased antioxidant oxidase content regulated HFHS diet-induced insulin resistance, offers the possibility that the CLP can be used as a treatment for diabetes.

The Polysaccharide (DAP) from the roots of *Dipsacus asper* has been used in STZ-induced diabetic rats. After administration with DAP, the fasting blood glucose and glycosylated haemoglobin (HbA1c) levels were decreased, but the body weight increase in STZ-induced diabetic rats. Moreover, advanced glycation end products-receptor accumulation were nearly reversed to the normal levels along with inhibition of AGE-RAGE expression in STZ-treated diabetic rat by DAP (Li T. et al., 2019).

The *Ophiopogon japonicus* polysaccharide (OJP) exhibited antioxidant effects by efficiently diminishing the levels of free radicals (DPPH and ABTS), markedly enhancing the NO production, and diminishing the mRNA expressions of iNOS, COX2 in nickel-induced RAW264.7 cells (Chen et al., 2013; Lin et al., 2020). Moreover, with OJP treatment, the levels of TG, TC, HDL-C and LDL-C were obviously reversed in diabetic rats. Altogether, these results suggest that OJP could be a natural antioxidant and can improve diabetes (Chen et al., 2013).

The polysaccharide isolated from *Rehmannia glutinosa* (RGP), as a main active ingredient exhibited hypoglycemic effects (Zhou et al., 2015a). In STZ-induced mice, RGP dispiayed anti-oxidative activities by elevating serum GPx and SOD levels, whereas decreasing blood levels of IL-6, MDA, TNF-α, and monocyte chemoattractant protein-1 (MCP-1) (Zhou et al., 2015a). Moreover, with RGP administration, high levels of TG, TC, LDL-C were reversed and HDL-C increased in diabetic mice.

Polysaccharides obtained from guava (*Psidium guajava* L.) Leaves (GLP) showed antioxidant and antidiabetic activities. In a STZ + HFD induced diabetic mice. These polysaccharides exhibited good OH, ABTS free-radical, and DPPH scavenging abilities, and significantly ameliorated fasting blood sugar, Total cholesterol (TC), MDA, creatinine, and Triglycerides (TG) levels. Meanwhile, it significantly increased SOD enzyme activity (Luo et al., 2019). Thus, GLP might be a promising candidate for the development of a new anti-diabetic or antioxidant agents. However, its clinical efficacy needs to be further verified.

Holothurian glycosaminoglycan from *Apostichopus japonicus* (AHG) can suppress hepatic glucose production for the development of an anti-hyperglycemic agent. In HFD-fed mice, AHG supplementation apparently reduced conventional index, such as body weight, blood glucose level, and serum insulin content. In same model, AHG elevated phosphorylation levels of insulin receptor substrate-1(IRS1), Akt, and AMPK levels that were decreased by HFD treatment (Chen Y. et al., 2019).

The *Pueraria lobata* polysaccharide (PLP) improved diabetic by scavenging blood glucose levels and alleviating the lipid metabolic function of db/db mice. In particular, PLP boosted the expression of G6PC, FOXO1, PEPCK, SREBP-1, and ACC, whereas diminished the mRNA expression of antioxidant-related indicators, such as glycogen synthase, phosphatidylinositol-3-kinase, Akt2, PPARa, and glucose transporter 2 (GLUT2) in the liver of db/db mice (Luo et al., 2021).

*Lycium barbarum* polysaccharides (LBPs) extracted from L. barbarum, markedly increased insulin secretion and insulin sensitizing activities to improve insulin resistance. They also regulated glucose metabolism through hypoglycemic and antioxidative activities to relieve diabetes (Li, 2007; Cheng et al., 2015; Shi et al., 2017). Compared with the diabetic group, LBPs promoted the production of antioxidant enzymes such as SOD, CAT, and GPx, while down-regulating Caspase-3 expression and up-regulating the ratio of Bcl-2/Bax in STZ-included diabetes mice (Shi et al., 2017). Simultaneously, LBPs activated the PI3K/Akt/mTOR pathway, and Nrf2/HO-1 pathway, whereas inhibited the AGEs-RAGE signalling pathway to mitigate OS (Li, 2007; Tian et al., 2019).

*Euryale ferox* polysaccharides (EFP) exhibited protective effects against alloxan-induced oxidative injury in mice. Furthermore, EFPPs can enhance CAT, SOD and GSH-Px expression and scavenging of MDA content in the liver and kidney of alloxan-induced hyperglycemic mice. In addition, EFPP was beneficial enhancing oral glucose tolerance, and hepatic glycogen content, but reversed blood glucose levels and relieved diabetes (Wu C. Y. et al., 2017).

*Physalis alkekengi* polysaccharides (PAP) have been reported to have an antidiabetic effect in mice. The PAP intervention decreased blood glucose and glycated serum protein levels. Moreover, PAP protected and reversed β-cell necrosis and upregulated the mRNA expression of PI3K, GLUT4, and Akt in alloxan-induced diabetic mice (Guo et al., 2017). Consequently, PAP could be explored as a potential for the development of a new treatment diabetic agent.

*Schisandra sphenanthera* polysaccharides (SSPs) regulated hyperglycemia, improved the glucose tolerance, reduced FBG, and elevated the levels of FINS and the value of ISI in STZ induced rats. After SSPs administration, MDA levels were also repressed and SOD, GSH-PX and CAT activities were enhanced (Niu et al., 2017).

The polysaccharide from Moutan Cortex (MC-P) ameliorates diabetes by down-regulating the AGEs pathway. It also decreased ROS production induced by AGEs in HG- and STZ-induced rats and the AGEs-stimulated human umbilical vein endothelial cells. Moreover, MC-P can regulate the production of inflammatory factors (VCAM-1 and ICAM-1) and improve TGF-β1 levels (Lian et al., 2021).

*Arctium lappa* polysaccharides (ALP) displayed anti-diabetes effect by regulating lipid metabolism and OS through the PKC/NF-κB pathway in diabetic rats. Administration with these polysaccharides ALP *in vivo*, led to upregulation of antioxidant enzymes such as SOD and GSH-Px, whereas a decrease in MDA levels decreased in the rat livers. Moreover, the expression of P-select, PKC-α, and PKC-β was obviously decreased. Meanwhile, the PKC/NF-κB signalling pathway was inhibited (Li X. et al., 2019).

The *Anoectochilus roxburghii* polysaccharide (ARP) has been reported to regulate OS. After the ARP intervention in HFD-induced male C57BL/6 mice for 12 weeks, the fatty acid pathway regulating lipogenesis was inhibited, the levels of antioxidant enzymes, such as GPX, glutathione S-transferase (GST) and SOD were increased, and MDA levels were decreased. Furthermore, the Nrf2-mediated phase II enzyme system (Nrf2/HO-1/NQO1) was activated to improve OS. Moreover, the mitochondrial complex and PGC-1α were activated to promote mitochondrial function. Furthermore, ARP also had some effects on reducing ROS levels and increasing ATP levels, thereby significantly improving diet-induced OS (Chen et al., 2021).

*Buddleja officinalis* (BOP) polysaccharides could improve diabetes by improving levels of blood glucose, blood lipids, and insulin and further improving CD34 expression. Furthermore, BOP diminished ROS levels, whereas promoted SOD, NQO1, Nrf2, and HO-1 expression levels in db/db mice to ameliorate diabetes (Zhu et al., 2022).

*Opuntia milpa alta* polysaccharides (MAPs) contain xylose (23%), arabinose (36%), and galactose (15%) and have been reported exhibit anti-diabetic effects. *In vitro* research, treatment with MAPs restored oxidative enzyme activities and cell viability improved NO, MDA and ROS, and regulated antioxidant PARP and Nrf2 pathways in alloxan-induced INS-1 cell. Moreover, MAPs regulated proteins related to apoptosis, such as the expression of Bcl-2, Bax, caspase-3, and caspase-9 (Li W. et al., 2020). These findings indicated that MAPs has the potential to regulate OS.

*Astragalus mongholicus* Bunge polysaccharides (APS) also repressed apoptosis by enhancing Bcl-2 levels and diminished Bax, caspase-3, and caspase-9 levels (Chang et al., 2018). In both STZ-induced diabetic mice and nondiabetic SOD2+/−mice, APS reduced the apoptosis and enhanced the proliferation of cardiac stem and progenitor cells (CSPCs). Furthermore, APS enhanced SOD2 protein levels and enzyme activities, and downregulated ROS formation and oxidative damage of CSPCs (Chen et al., 2018). In HG-induced or SOD2-silenced H9C2 cells, APS improved cell apoptosis and higher levels of ROS, and regulated the levels of oxidative stress injury indicators 8-OH-dG and nitrotyrosine (Sun et al., 2019). More polysaccharides from medicinal plant with antioxidation and anti-diabetic effects show in Table 1.

**TABLE 1:** Polysaccharides from medicinal plant with antioxidation and anti-diabetic effects.

Polysaccharide source	Monosaccharides composition	Models	Administration	Mechanism	Positive control	Reference
*Abelmoschus esculentus* (L.) Moench	Man: rha: glucuronic acid, galactosal acid, gal: ara = 3.4:3.76:24.19:6.27:8.73:3.13	HFD combined with STZ induced mice	200 or 400 mg/kg	↓: TG, TC, LDL-C; MDA, body weight, and ROS	Metformin	Liao et al. (2019a)
				↑: HDL-C, SOD, GSH-Px and CAT, Nrf2, HO-1, SOD2, PI3K/AKT/GSK3β pathway		
*Abelmoschus esculentus* (L.) Moench	Rha: Gal: Gal A = 1.87:3.58:1.00	HFD and STZ induced mice	200, 400 mg/kg	↓: ROS, MDA, caspase-3, and Bax	Metformin	Liao et al. (2019b)
				↑: SOD, Bcl-2 and CAT, Nrf2, HO-1, SOD2, AMPK/AMPK pathway		
*Cyclocarya paliurus* (Batal.) Iljinskaja	Glc: ara: gal: man: xyl: rha: galacturonic acid: glucuronic acid: fuc: rib = 27.90:9.68:7.67:1.93:1.67:1.26:0.72:0.66:0.17:0.16	STZ induced diabetic rats	1, 10 and 100 mg/kg/·d for 12 weeks	↑: SOD, CAT, GSH-Px, and GSH	Metformin hydrochloride	Xia et al. (2020)
				↓: AGEs and TGF-β1		
*Cyclocarya paliurus* (Batal.) Iljinskaja	Rha: ara: xyl: man: glu: gal	STZ induced rats	1, 10, 100 mg/kg/d for 12 weeks	↑:SOD, CAT, GSH-Px, GSH		Zhang et al. (2021)
				↓: AGEs, ROS, and TGF-β1		
*Fructus Corni*	Man: gal A, glc: xyl: gal: ara = 0.95:0.08:3.17:1.88:1.23:0.25	STZ-induced diabetic rats	800 mg/kg on 42nd day	↑: ACT, SOD, and GSH	Metformin hydrochloride	Wang et al. (2019a)
*Astragali radix*	Glc, gal A, ara, gal, glu A xyl	STZ induced male C57BL/6J mice	400 mg/kg/d	↑: SOD, GSH, CAT		(Chen et al., 2022) (Chen et al., 2018)
				↓: MDA levels		
*Hedysarum alpinum* L.		ob/ob mice	50, 100, and 200 mg/kg/d	↓: Keap1 signalling	lipoic acid	He et al. (2022)
				↑: Nrf2 signalling		
*Hedysarum alpinum* L.		HG induced Schwann cells	30, 60, 120, and 240 mg/L	↓: MDA		He et al. (2022)
				↑: GCLC, GR		
*Morus alba* L.		STZ+HFD induced rats		↓: MDA		Liu et al. (2017)
				↑: SOD, CCO and SDH		
Mulberry leaves		STZ induced diabetic rats	50–200 mg/kg for 5weeks	↓: MDA		Li et al. (2022)
				↑: SOD		
*Dendrobium officinale* Kimura & Migo	D-Glcp: D-Manp = 1.00:4.41. 190 kDa	male wistar rats	20, 40, 80, and 160 mg/kg/d for 8 weeks	↑: SOD, CAT, and T-AOC	Metformin	Yang et al. (2020a)
*Polygonatum sibiricum* Redouté		STZ induced rats	200, 400 and 800 mg/kg	↑: insulin		Wang et al. (2017d)
				↓: MDA, blood glucose		
*Polygonatum sibiricum* Redouté		HG-induced ARPE-19 cells		↓: MDA, ROS, bcl-2		Wang et al. (2022)
				↑: SOD and GPx, Bax, caspase-3 activity, Nrf2/HO-1 pathway		
*Polygonatum sibiricum* Redouté		1 μmol/L insulin and 25 mmol/L glucose induced 3T3-L1 adipocytes	50, 100, 250, 500 μg/mL	↑: Nrf2,HO-1		Cai et al. (2019)
				↓: IL-6, IL-1βand TNF-α		
*Codonopsis lanceolata* (Siebold & Zucc.) Benth. & Hook.f. ex Trautv	Rha: Ara:xyl:man: gal: glc: Gal A: glu A = 0.17:1:0.12:0.05:0.26:2.32:0.19:0.95	HFHS diet-fed mice	100 mg/kg	↓: MDA		Zhang et al. (2020)
				↑:GSH/GSSG ratio, SOD, CAT, Nrf2 signalling		
*Dipsacus asper* Wall. ex DC.	D-glu	STZ-induced diabetic rats	100 and 300 mg/kg for 4 weeks	Downregulated oxidative stress and glycation end products-receptor	Metformin	Li et al. (2019a)
*Ophiopogon japonicus* (Thunb.) Ker Gawl	Ara: Glu: gal = 1:16:8	STZ induced mice	150 mg/kg/d for 28 days	↑: GPx and SOD	Metformin	Chen et al. (2013)
*Ophiopogon japonicus* (Thunb.) Ker Gawl		RAW264.7 cells induced by nickel	10, 50, and 100 μg/mL for 24 h	↓: DPPH, ABTS, NO, iNOS, COX2		Lin et al. (2020)
*Rehmannia glutinosa* (Gaertn.) DC.	Rha: ara: man: glc: gal = 1.00:1.26:0.73:16.45:30.40; 63.5 kDa	STZ-induced diabetic mice	20, 40 and 80 mg/kg/day	↑: GPx and SOD activities	Metformin	Zhou et al. (2015a)
				↓: MDA, IL-6, TNF-α, and MCP-1		
*Psidium guajava L*	polysaccharides (≥80.99 kDa) and low molecular weight polysaccharides (3.64 kDa)	STZ and HFD induced mice	100 mg/kg, 200 mg/kg/d	↑: GPx and SOD activities	Acarbose	Luo et al. (2019)
*Apostichopus japonicus*	→4GlcA (Fuc2S,4Sα1→3)β1→3GalNAc4S6Sβ1→	HFD for 12 weeks	50 mg/kg/day	↑: P- IRS1, Akt, and AMPK	Metformin	Chen et al. (2019b)
*Pueraria lobata* (Willd.) Ohwi (Fabaceae)		db/db mice	100 or 200 mg/kg for 6 weeks	↓: PEPCK, G6PC, FOXO1, SREBP-1, and ACC	Rosiglitazone	Luo et al. (2021)
				↑: GS, Akt2, PI3K, GLUT2, PPARa, and LDLR		
*Lycium barbarum L*	ara, glc, gal, man, rha, xyl, gal A	STZ-induced diabetic mice	50, 100, 200 mg/kg for 30 days	↓: MDA		Li (2007)
				↑: SOD		
		STZ-included mice	10, 20, and 40 mg/kg	↓: Caspase-3	Sildenafil citrate	Shi et al. (2017)
				↑: Bcl-2/Bax		
*Euryale ferox Salisb.*	Man, GlcA, Rha, Glc, Gal and Ara at a molar ratio of 0.12:0.01:9.57:0.41:1.00:0.24	alloxan-induced hyperglycemic mice	100,200,400 mg/kg/d	↑: CAT, SOD, oral glucose tolerance, hepatic glycogen content and GSH-Px	metformin hydrochloride	Wu et al. (2017a)
				↓: MDA, glucose level increase		
*Physalis alkekengi* var. franchetii Makino		Alloxan-induced hyperglycemic mice	200, 400, and 800 mg/kg	↑: PI3K, Akt and GLUT4 mRNA; Hypoglycaemic; protecting and reverse β-cells from necrosis	Acarbose	Guo et al. (2017)
*Schisandra sphenanthera* Rehder & E.H.Wilson	Rha:Ara:Man:Gal:Glc = 13.52:5.69:3.92:41.28:35.59	STZ induced rats	100,200,400 mg/kg/d	↑: GLUT-4; mitigate the insulin resistance; hpyerglycemic; improving the glucose tolerance	glibenclamide	Niu et al. (2017)
*Paeonia × suffruticosa Andrews*	D-glucose: L-arabinose = 3.31:2.25	high-sugar diet and STZ treatment rats	80, 160 mg kg	↓: serum creatinine, AGEs/RAGE		Lian et al. (2021)
*Moutan Cortex*		200 μg/mL AGEs induced human umbilical vein endothelial cells for 24 h	64.5 μg/mL	↓: ROS	Aminoguanidine	Lian et al. (2021)
*Arctium lappa* L	Gal: Man: Glc: Rha: Xyl: GlcN: GlcA = 6:6.4:26.5: 3.3:26.2:7 2	high-sugar, HFD, and STZ	100, 200 mg/kg for 40 days	↓: PKC expression, MDA, and NF-κB pathway		Li et al. (2019b)
				↑: SOD and GSH px		
*Anoectochilus roxburghii* (Wall.) Lindl		HFD induced mice	100, 200, and 400 mg/kg	↑: GST, GPX, GST and SOD		Chen et al. (2021)
				↓: MDA, ATP		
*Buddleja officinalis Maxim*	glucose, galactose, fucose, glucuronic acid, and galacturonic acid in a ratio of 6.75 : 3.33 : 1.79 : 1.42 : 1.00	db/db mice	40,80,160 μg/mL	↑: HO-1, NQO1, SOD and Nrf2/ARE signalling pathway		Zhu et al. (2022)
				↓: CD34, ROS		
*Opuntia milpa alta*	Xylose: arabinose: galactose = 23:36:15	alloxan-treated INS-1 cells	50, 100, and 200 μg/mL	↑: Bcl-2, MDA, and NO		Li et al. (2020b)
				↓: Bax, ROS, caspase-3, caspase-9		
*Astragalus mongholicus* Bunge	D-glu: D-gal: L-ara = 1.75:1.63:1	HG induced H9C2 cells for 24 h	0.1,1,10,100 μg/mL	↑: SOD2, GSH-Px		Sun et al. (2019)
				↓: ROS, MDA, and NO		

#### 3.1.2 Food

The *Siraitia grosvenorii* polysaccharide (SGP-1) alleviated inflammation by reducing the cytokines IL-6 and TNF-α. Moreover, it regulated oxidation by stimulating SOD production of and diminished MDA in DN mouse models (Gong et al., 2021).

Polysaccharides obtained from the dried pumpkin pulp (PPs) have hypoglycemic effects (Wang S. et al., 2017). After PPs treatment OS was improved through the upregulation of the Nrf2, HO-1, NOS, MDA and PI3K levels (Chen X. et al., 2019). They also can reduce TG, TC, and LDL-C levels and improve HDL-C, SOD, PI3K levels in the hypoglycemic mechanism (Chen X. et al., 2019).

The *Ficus pumila Linn* polysaccharide (FPLP) improved glycogen metabolism by activating the phosphoenolpyruvate carboxykinase and glucose-6-phosphatase expression, regulating the IRS-1/PI3K/Akt/GSK3β/GS and AMPK/GSK3β/GS signalling pathway and glucokinase in C57BL/KsJ db/db mice (Wu J. et al., 2017). Mechanistically, the PI3K/Akt pathway and AMPK signalling pathway are closely related to the oxidation reaction process of the body. This suggests that FPLP may have the potential to ameliorate diabetes by regulating OS (Wu J. et al., 2017).

The peach [*Prunus persica* (L.)] tic gum polysaccharide (PGP) could restore the postprandial blood glucose levels in STZ-induced diabetic mice by recovering pancreaislets, and activating the expression of HO-1 and insulin, which are all beneficial for improving diabetes (Wang et al., 2017c).

The mulberry fruit polysaccharide (MFP) also possesses numerous bioactivities. In STZ induced diabetic mice deal with STZ, MFP can promote pancreatic β cells proliferation and thus increase insulin secretion. Moreover, MFP can improve diabetes by regulating OS, which is mainly manifested in inhibiting MDA content, and increasing SOD, GPx, and CAT levels (Chen et al., 2017).

The polysaccharide from the sweet potato (SPP) significantly alterated the lipid metabolism index (TC, TG, and LDL), while remarkably decreasing HDL levels in STZ-induced rats. Compared with the diabetic rats, the level of MDA decreased along with an increase in SOD, GSH-Px, GSH and CAT levels after SPP treatment (Yuan et al., 2017).

*Momordica charantia* L. has been reported to exhibit antiobesity and antidiabetes activities (Zhang et al., 2019). In an *in vitro* study, this bitter gourd polysaccharide improved diabetes mice by enhancing the concentration of CAT, SOD, and GSH-px antioxidant enzymes and decreasing the content of MDA in mice (Zhang et al., 2019).

The *Dipsacusa sper* polysaccharide (DAP) regulated hyperlipidemia, downregulated the formation of the advanced glycated end-product receptor (AGE-RAGE), and improved OS in STZ-treated diabetic rats (Li et al., 2019).

A novel polysaccharide (RTFP-3), extracted from *Rosa roxburghii* fruit, has been found have antioxidant activity. In H_2_O_2_-induced INS-1 cells, RTFP-3 possessed higher protective and suppressive activities against H_2_O_2_-induced apoptosis of INS-1 cells in comparison with normal. Additional, after treatment with RTFP-3, ROS was decreased in INS-1 cells, which indicated that RTFP-3 via attenuating oxidative stress to prevent dibaetes (Wang et al., 2019). More polysaccharides from foods with antioxidation and anti-diabetic effects show in Table 2 **.**

**TABLE 2:** Polysaccharides from foods with antioxidation and anti-diabetic effects.

Polysaccharide source	Monosaccharides composition	Models	Administration	Mechanism	Positive control	Reference
*Siraitia grosvenorii*	Ara: Rib: GalAc: Gal: Man: Glc = 1.00:1.72:90: 2.24:3.64:3.89: 22.77	HFD and HSD induced mice	50, 100, and 200 mg kg/d	↓: IL-6, TNF-α and MDA		Gong et al. (2021)
				↑: SOD		
Pumpkin		STZ + HFD induced rats		↓: blood glucose, insulin, TC, TG, LDL-C, and MDA		Ti et al. (2022)
				↑: HDL-C, SOD, and CAT		
pumpkin	Man: rib: glc: glu A:gal A: glc: gal: xyl: fuc = 142.92:42.89: 1.03:17.83:2.6: 125.75:0.85 : 112.34:73.25	HFD and STZ to induce T2DM	400 mg/kg/day	↑: Nrf2; HO-1, PI3K		Chen et al. (2019a)
*Ficus pumila Linn*	A linear (1,4)-α-D-galacturonic acid binding 1.30% branched chain hexenuronic acid with 23.34% methyl esterification	C57BL/KsJ db/db mice	100,200 mg/kg/d	activated the IRS-1/PI3K/Akt/GSK3β/GS and AMPK/GSK3β/GS signalling pathway	rosiglitazone	Wu et al. (2017b)
Peach	Ara:Xyl:Gal = 5.98:1:3.55	STZ-treated mice	200, 400 and 800 mg/kg	↓: blood glucose	Metformin hydrochloride	Wang et al. (2017c)
				↑: pancreatic duodenal homeobox-1, insulin and hexokinase1		
Mulberry fruit	Ara:Gal:Glc:Xyl:Man = 19.19:31.4:26.31:5.98:7.12	STZ-induced diabetic mice	200,600,1000 mg/kg/d	↓: MDA; α-amylase, and α-glucosidase	metformin	Chen et al. (2017)
				↑: SOD, GPx, CAT, insulin secretion ameliorate insulin resistance; PI3 K/Akt pathway activators		
Sweet potato	Rha:Gal:Glc = 3.2:1.6:6.5	STZ rats	100 mg/kg/d	↓: MDA	glibenclamide	Yuan et al. (2017)
*Momordica charantia* L	Rha: ara: xyl: man: glc: gal: fuc = 15.7,23.6,11.9,.6,31.22,12,1.1	STZ-induced DM mice	1,500 mg/kg	↑: CAT, SOD, GSH-px	metformin	Zhang et al. (2019a)
				↓: MDA		
*Rosa roxburghii* fruit	Ara: gal: fuc: glu:man: xyl = 37.20: 34.14: 18.30: 10.02:0.15:0.17	H_2_O_2_-induced INS-1 cells	0.25, 0.5, 0.75, 1, 1.5, 2 mg/mL	↓: ROS; mitochondrial damage		Wang et al. (2019b)
				↑: caspase-3, caspase-8, and caspase-9		

### 3.2 Polysaccharides derived from Microorganisms

The *Agrocybe Cylindracea* polysaccharide (ACP) can decrease blood glucose levels, heal liver and colon injuries, and repress inflammation (Wu L. et al., 2022). It also can increase SOD, GSH-Px, and CAT levels and improve lipid metabolism in HFD combined STZ induced mice (Sun et al., 2022).

*Cordyceps militaris* (CM), as a TCM, has been reported have anti-diabetes effects (Chen, 2015). In animal experiments, it was found that CM polysaccharide (CMP) could downregulate the contents of lipid peroxidation, blood lipid and blood sugar. Besides, it could improve insulin resistance and blood glucose levels. In particular, CMP increased the activities of antioxidant enzymes, such as glutathione peroxidase (GSH-px), superoxide dismutase (SOD), and catalase (CAT) in HFD and STZ induced mice (Zhao et al., 2018). Similarly, in another research, a polysaccharide-enriched fraction obtained from CM also produced hypoglycemic effects in STZ-induced rats (Zhang et al., 2006).The anti-diabetic effects of C. cicadae polysaccharide (SHF) were evaluated in alloxan-induced diabetic rats. SHF administered repressed the body weights of rats. Additionally, GSH, SOD, and HDL levels were upregulated, whereas MDA, urea, LDL, TG, CREA, TC, ALP, AST, and ALT levels were downregulated with SHF treatment (Zhang et al., 2018).

Mushroom (*Cynomorium coccineum* L.) polysaccharides (MPs), derived from the mycelium and the fermentation broth, have a role in anti-diabetes treatment (Zhao et al., 2014; Chen, 2015; Liu et al., 2022). MP can enhance the levels of antioxidant enzymes such as SOD, GSH-Px, and CAT in diabetes models *in vivo* (Chen, 2015; Zhang et al., 2015; Zhang et al., 2017; Liu et al., 2022). The polysaccharide hispidin from *Phellinus linteus* (Berk. and M.A. Curtis) improved the β-cell activities by inhibiting H_2_O_2_ induced apoptosis and improving insulin levels (Maity et al., 2021).

*Ganoderma lingzhi* polysaccharides (GLPs) exhibited outstanding antioxidant, hypoglycemic, and hypolipidemic activities in STZ-induced T2DM rats (Liu, 2019; Xu et al., 2019; Chen et al., 2020). More data on the role of MP in diabetes treatment can be obtained from another review (Arunachalam et al., 2022). Furthermore, GLP improved the OS and inhibited apoptosis by increasing the expression of TGF-β1, GSH, SOD, and NOS and suppressing the phosphorylation of MDA, JNK, eNOS and ERK in DM rats (Yao et al., 2022). In addition, GLP-treatment reversed the levels of fasting plasma lipids, hepatic lipid accumulation, and higher blood glucose levels (Li et al., 2020), increased SOD, CAT, and GSH-Px expression, as well as promoted Nrf2 and HO-1expression in db/db mice, by contrast, the content of MDA and TNF-a decreased in these mice.

*Auricularia auricular* (L.et Hook.) Underw polytricha (AAP) are popular edible fungi. At a low dose (100 mg/kg/day), AAP generally increased the vitality of antioxidant enzymes in STZ-induced diabetes mice, and might regulate the NF-κB pathway and the associated signalling pathway to alleviate diabetes (Xiang et al., 2021).

*Lentinus edodes* polysaccharides (LEPs) have been found to exhibit hypoglycemic ability by improving the Nrf2/HO-1 pathway. The LEP can increase SOD and GSH activity, as well as reduce MDA levels in STZ induced DM mice (Gong et al., 2022). Furthermore, in an *vivo* study, LEP reduced cell damage by increasing SOD activity, decreasing ROS content, and revising HG-induced MDA levels in MIN6 cells (Cao et al., 2019). In addition, LEP increased the viability of HG-induced human umbilical vein endothelial cells, decreased ROS production, and reversed the inhibition of α-glucosidase activity. It especially inhibited AGE formation in cells (Cao et al., 2020).

The polysaccharide from the caps of *Suillellus luridus* (SLP) exhibited excellent antidiabetic activities by improving Nrf2/HO-1-mediated OS (Liu et al., 2020a) in the STZ-induced diabetic mice. SLP was composed of galactose (gal), glucose (glu), arabinose (ara), and mannose (man). It had a backbone principally composed of 1,3 linked α-D-Galp, 1,3 linked β-D-Glcp and 1,6 linked β-D-Glcp with the branches mainly composed of T-linked α-D-Galp, 1,3 linked α-L-Arap, 1,3 linked β-D-Glcp, and 1,3 linked α-D-Manp. Moreover, SLP regulated the mRNA levels and protein expression in NF-kB signaling pathways. In short, SLP can be treated as a potential agent for preventing and treating diabetes via regulating oxidative stress (Liu et al., 2020a).

Polysaccharides from *Armillariella tabescens* mycelia diminished the levels of blood glucose, and repressed OS cytokine. They increased SOD and GSH concentrations, and decreased MDA production in diabetic mice to improve T2DM (Yang et al., 2020).

Polysaccharides separated from *Inonotus obliquusvia* (IOP) modulated OS in STZ-induced mice (Wang et al., 2017). Simultaneously, it remarkably regulated DPPH scavenging activity and ferric-reducing power in H_2_O_2_-induced hepatic L02 cells (Wang et al., 2018).

Polysaccharides from the mycelia of *Coprinus comatus* (CMP) reduced insulin resistance in STZ-induced mice models. It is mainly manifested in the many aspects, they improved energy metabolism, suppressed OS and inflammation, and modulated the Wnt-1/β-catenin and PTEN/PI3K/Akt pathways (Gao et al., 2021).

Extracellular polysaccharides (EPS) from *Pleurotus eryngii* SI-04 exhibited anti-glycated activities in STZ-regulated diabetic mice. EPS significantly suppressed GLU levels; decreased BUN, ALB, CRE and UA levels; downregulated serum lipid levels; modified the content of antioxidant enzymes, such as CAT, GSH-Px, SOD, and MDA (Zhang et al., 2018).

*Paecilomyces hepiali* polysaccharides (PHP) exerted anti-diabetic properties by regulating Nrf2-meadited NF-κB signalling in db/db mice. They not only decreased ROS and MDA concentrations, but also increased GSH-Px, CAT, and SOD contents in the serum and kidney (Hu et al., 2020).

*Grifolafrondosa frondosa* polysaccharides exert hypoglycemic, anti-diabetic, and nephritic properties by modulating the content of antioxidant enzymes in HFD- and STZ-induced diabetic rats. When CAT, SOD, and GSH-px levels increased, ROS levels decreased in the serum (Kou et al., 2019).

*Pholiota nameko* polysaccharides (PNPs) exhibited antiglycation ability by improving methylglyoxal -induced Hs68 cell damage. Preprocessing of Hs68 cells with PNPs increased the cell survival rate and inhibited intracellular ROS content. Furthermore, PNPs significantly decreased AGEs and inhibited ROS production, thus alleviating cell damage and OS (Lin et al., 2021). Representative polysaccharides from microorganisms with antioxidation and anti-diabetic effects show in Table 3.

**TABLE 3:** Polysaccharides from microorganisms with antioxidation and anti-diabetic effects.

Polysaccharide source	Monosaccharides composition	Models	Administration	Mechanism	Positive control	Reference
*Agrocybe Cylindracea*	Man, rib, rha, glu A, gal A, glu, gal, xyl, ara, and fuc	HFD and STZ-induced mice	100, 200, and 400 mg/kg/d for 4 weeks	↓: Blood glucose, liver and colon injuries, and inflammation		Sun et al. (2022)
				↑: SOD, GSH-Px, CAT, and lipid metabolism		
*Cordyceps sinensis*	Fuc: rib: ara: xyl: man: gal: glc = 1.23:0.57: 0.29:2.12: 2.73:4.66: 88.4	HFD and STZ induced mice	100 and 400 mg/kg, for 4 weeks	↑: GSH-Px, SOD, and CAT		Zhao et al. (2018)
				↓: MDA, LDL, TC, TG, urea, CREA, ALT, AST, and ALP		
*Cordyceps cicadae*		alloxan monohydrate induced rats	100, 200 and 400 mg/kg for 30 days	↑: HDL, SOD, and GSH	glibenclamide	Zhang et al. (2018c)
				↓: TC, TG, LDL, MDA, urea, CREA, ALT, AST, and ALP		
Cynomorium coccineum *L*		Male SD rats	1.0, 2.0 g/kg for 4 weeks	↓: α-glucosidase, blood glc, MDA, LDL-C, TC, and TG		Zhang et al. (2017)
				↑: SOD, GSH-Px, and HDL-C		
*Phellinus linteus*		alloxan-induced mice	100 mg/kg	↓: blood glc level	metformin	Zhao et al. (2014)
Mycelium zinc		STZ induced mice	800, 400, 200 mg/kg	↑:GSH-Px, SOD, CAT, and HDL-C		Zhang et al. (2015)
				↓:MDA, ALT, AST, BUN, CRE,TC,ALB, LDL-C, and VLDL-C		
*Ganoderma lucidum*	Rha:xyl:fru: Gal:man:glu = 0.793: 0.964: 2.944: 0.167: 0.384: 7.94	HFD induced male db/db mice	100 mg/kg/d	improving the Nrf2/HO-1 signalling pathway	metformin	Li et al. (2020a)
*Auricularia auricular* (L.et Hook.) Underw	Fuc, glu, gal, xyl, Rha, man; 17.1 kDa	STZ-induced diabetic mice	100, 300 mg/kg, For 4 weeks	↓: Blood glucose, TNF-α, and MDA		Xiang et al. (2021)
				↑: serum insulin, SOD		
*Lentinus edodes* (Berk.) sing	Rha: Fuc: Ara: GLC-UA:Gal: Man: Glc = 1 : 6.13 : 1.28 : 1.79 : 20.62 : 4.74 : 842.17	STZ mice	50, 100, and 200 mg/kg	↑: Nrf2/HO-1 pathway		Gong et al. (2022)
*Suillellus luridus*	Gal: Glc: Car: Man = 44.9:27.6:14.7:12.8; 9.4 kDa	STZ-induced diabetic mice	100 mg/kg/d for 30 days	↑: Hepatic glycogen, CAT, insulin, SOD, GSH-Px, and HDL-C	glibenclamide	Liu et al. (2020a)
				↓:blood glucose, TC, TG, and LDL-C		
*Suillellus luridus*	Fuc: glc: gal: xyl: rha: man; 173 kDa	STZ-induced diabetic mice	100,300 mg/kg for 4 weeks	↓: Blood glucose and MDA	glibenclamide	Zhang et al. (2018b)
				↑: serum insulin, CAT		
*Armillariella tabescens* mycelia	Man, ara, fuc	HFD and STZ induced mice	200, 400 mg/kg	↑: SOD, GSH		Yang et al. (2020b)
				↓: ROS, LPO and MDA		
*Inonotus obliquusvia*		H_2_O_2_-induced hepatic L02 cells	50, 250 and 500 μg/mL	↓: DPPH, ferric reducing power	Vc	Wang et al. (2018)
*Coprinus comatus mycelium*	Gal,α-pyranose; 495.8 kDa	HFD-STZ fed mice	100, 200, 400 mg/kg/d	modulating the PTEN/PI3K/Akt and Wnt-1/β-catenin pathways	metformin	Gao et al. (2021)
*Pleurotus eryngii* SI-04	Ara: Man:Gal:Glu = 1:1.7:1:4.3	STZ induced mice	600 and 300 mg/kg	↓: GLU, ALB, BUN, CRE, UA levels, TC, MDA, TG, VLDL-C and LDL-C	glibenclamide	Zhang et al. (2018a)
				↑:GSH-Px, SOD, and CAT		
*Paecilomyces hepialid*	D-Xylose (D-Xyl), D-Mannose (D-Man), D-Glc and D-Gal	db/db mice	10, 20, and 40 mg/kg	↑: CAT, GSH-Px, and SOD	metformin	Hu et al. (2020)
				↓: MDA, ROS		
*Grifola frondosa* (Dicks.) Gray	Glc:Man:Gala:Xyl:Ara:Rha:Ribose = 26.74:22.79:16.76:16.02:14.29:2.05:1.35/Ribose:Ara:Xyl = 74.73:14.20:11.08/Rha:Ara:Xyl:Man:Glc:Gal = 4.74:5:1:3.42:31.29:6.89	STZ induced diabetic rats	200 mg/kg	↑: CAT, SOD, GSH-px	metformin	Kou et al. (2019)
				↓: ROS		
*Pholiota nameko*		methylglyoxal -induced Hs68 cell		↓: ROS, AGEs		Lin et al. (2021b)

### 3.3 Polysaccharides derived from marine life.

*Sargassum thunbergii* polysaccharides (STP), as a promising natural antioxidant and hypoglycemic agent, can be used to improve diabetes. They not only exhibited strong antioxidant and α-glucosidase inhibitory activities, but also improved the glucose uptake and radical-scavenging (DPPH, ROS) rates in insulin-resistant HepG2 cells (Ren et al., 2017).

The polysaccharide from *Laminaria japonica* (LGP) displays various pharmacological functions, especially in oxidative stress. LGP significantly improved mitochondrial dysfunction, leading to higher ATP and SOD content and lower ROS content. Furthermore, LGP upregulated Nrf2 pathway and repaired dysfunction in H_2_O_2_-induced β-cell (Wu et al., 2022).

### 3.4 Polysaccharides derived from animal

Field cricket (*Gryllus bimaculatus*), as an edible insect, has been reported to have multiple effects (Table 3). Field cricket glycosaminoglycan has regulated diabetes by significantly increased CAT, SOD and GSH-Px content in db mice (Ahn et al., 2020).

The *Misgurnus anguillicaudatus* polysaccharide (MAP) has a potential hyperglycemic effect (Table 3). It repressed the levels of inflammatory factor, such as IL-6, TNF-α, MDA, and MCP-1, whereas boosted SOD and GPx activities in STZ-induced mice (Zhou et al., 2015b). Representative polysaccharides from marine life and animal with antioxidation and anti-diabetic effects show in Table 4.

**TABLE 4:** Polysaccharides from marine life and animal with antioxidation and anti-diabetic effects.

Polysaccharide source	Monosaccharides composition	Models	Administration	Mechanism	Positive control	Reference
*Sargassum thunbergii*	Ara: gal: glu: xyl:man:gal A: glu A = 1.94: 30.7: 4.54: 23.2: 17.6: 8.11:13.9	Insulin-resistant HepG2 cells	0.1, 0.5, and 1.0 mg/mL) for 24 h	↓: α-glucosidase; improving the glucose uptake in insulin-resistant		Ren et al. (2017)
*Laminaria japonica*		MIN6 cells exposed 125 mM H2O2 for 2 h	25, 50, 100, or 200 mg/mL	↓: ROS		Wu et al. (2022b)
				↑: SOD, SIRT1-PGC1-α pathway, Nrf2		
Field cricket (*Gryllus bimaculatus*)	Rha: rib: ara: fru: glu = 81.10:7.16:5.91:1.91:1.62	Cg-m+/+Leprdb, heterozygous (db/+) and homozygotes (db/db)	5 mg/kg	↑: CAT, SOD and GSH-Px activities	metformin	Ahn et al. (2020)
*Misgurnus anguillicaudatus* (Cantor)	Gal:Fuc:man = 5:4:1	STZ induced diabetic mice	50, 100 and 200 mg/kg	↓: TNF-α, IL-6, MCP-1, and MDA	metformin	Zhou et al. (2015b)
				↑: SOD and GPx		

### 3.5 Characteristics of polysaccharides in improving oxidative stress in diabetes mellitus

**FIGURE 4:**
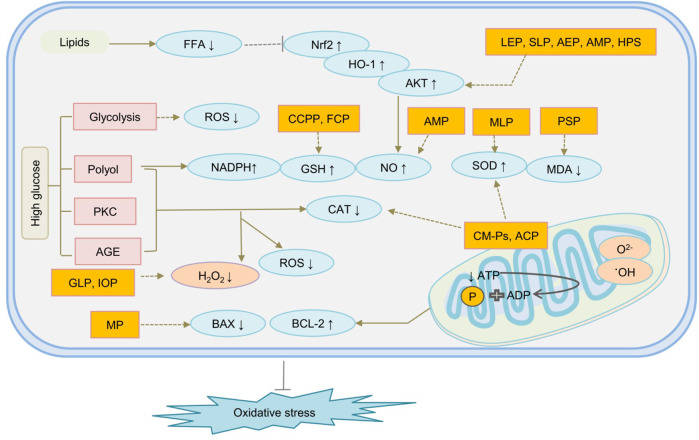
The mechanism of natural polysaccharides treat diabetes through oxidative stress is shown follows. ACP, Agrocybe Cylindracea Polysaccharide; AEP, Abelmoschus esculentus polysaccharide; AMP, Astragalus membranaceus polysaccharide; AMP, Astragalus membranaceus polysaccharide; CCPP, Cyclocarya paliurus polysaccharide; CM-Ps, acidic-extractable polysaccharides; FCP, Fructus Corni Polysaccharides; GLP, Ganoderma lucidum polysaccharide; HPS, Hedysarum polysaccharide; IOP, Polysaccharides separated from Inonotus obliquusvia; LEP, Lentinus edodes polysaccharides; MLP, mulberry leaf polysaccharide; MP, Mushroom polysaccharides; PSP, Polygonatum sibiricum polysaccharides; SLP, Suillellus luridus Polysaccharide; STP, Sargassum thunbergii Polysaccharides.

As we all know, excessive sugar intake is a contraindication in diabetic patients, which will cause increased insulin secretion, resulting in damage to islet beta cells, causing elevated blood sugar. But natural polysaccharides, as a special class of compounds, have been found not only to improve high blood sugar, but also to treat diabetes through other routes. Acting as an antioxidant, natural polysaccharides may control OS-induced diabetes mainly in two ways. They improved type 2 diabetes by inhibiting the production of reactive oxygen species, improving mitochondrial function impairment, and regulating signalling pathways such as oxidative pathway AGE, which is closely related to hyperglycemia patients. They may ameliorate OS by controlling the production of ROS, improving mitochondrial dysfunction of β cells, scavenge free radicals, or enhance antioxidant defense enzymes and their related pathways, such as Nrf2 pathway, oxygenase-1 (HO-1) pathway, Kelch-like ECH-associated protein 1 (Keap1) pathway, and antioxidant response elements (ARE) pathway to improve OS-induced insulin resistance (Nishikawa et al., 2000; Giacco and Brownlee, 2010; Yaribeygi et al., 2020). In addition, polysaccharides may ameliorate diabetes by regulating the cascade of molecular events in different metabolic pathways, such as glycolysis, hexosamine, PKC, polyols, and advanced AGE pathway. The mechanism of representative polysaccharides underlying diabetes treatment by OS is given in Figure 4.

### 3.6 Boundedness of polysaccharides in diabetes improvement

Although numerous natural polysaccharides have been shown to have a therapeutic effect on diabetes and oxidative stress inhibition, their pharmacological effects is closely linked to the structure of these compounds. Currently, the primary structure analysis is the main method used to determine the structure of natural polysaccharides, and the structure-activity relationship of natural polysaccharides cannot be accurately predicted. Moreover, the structural analysis of natural polysaccharides from food, marine organisms, and animals is relatively limited. The characterization of the advanced structure of natural polysaccharides is still in the exploration stage, and there are few studies on the functional fragments of natural polysaccharides that can clarify the activity orientation. Although many scholars have investigated the efficacy of natural polysaccharides in improving diabetic oxidative stress and attempted to speculate on the possible mechanism of action, there is a lack of innovation and breakthrough, and few in-depth studies have been conducted. Again, because the precise structure of polysaccharides is not clear, the application of polysaccharides is still only in animal or cell models, and there is not much clinical trial evidence. Whether the role of polysaccharides *in vivo* and *in vitro* models can fully reflect the efficacy of polysaccharides in diabetic patients in the future is a question worthy of consideration for every scholar.

## 4 Conclusion and future perspectives

More than 50 natural polysaccharides have been documented to improve oxidative stress, by regulating mitochondrial function, free radical levels, oxidase content, and Nrf2/HO-1, and AGEs/RAGE pathways in diabetes. However, intervention in diabetes and its complications is the result of multi-target, multi-pathway synergies. For example, polysaccharides extracted from fermented Momordica charantia significantly increased intestinal flora diversity and elevated the levels of short-chain fatty acids (SCFAs), thus exhibiting antidiabetic effects in HFD + STZ-induced rats (Gao et al., 2018). Furthermore, polysaccharides obtained from L. barbarum increased SCFAs levels, elevated gut microbiota diversities, and improved the intestinal barrier in HFD mice to mitigate diabetes (Yang et al., 2021). Bupleurum polysaccharides not only decreased blood creatinine, blood glucose, and urine albumin levels but also repaired the gut barrier and regulated inflammatory responses (Feng et al., 2019). Natural polysaccharides may also improve diabetes by regulating the function of glucose receptors. For example, SSP could promote GLUT-4 levels in BRL cells (Jin et al., 2016). Natural polysaccharides may have many potential antidiabetic activities waiting for us to explore. At the same time, we should also consider how to identify the key signalling pathways and core targets involved in the regulation of natural polysaccharides. It is worth exploring whether network pharmacology can be used to predict potential core targets of natural polysaccharides, and if the signalling pathway of significant enrichment of core targets can be used as the research pathway. Finally, animal experiments or *in vitro* cell experiments can be conducted to provide evidence.

The study of natural polysaccharides is currently limited to animal and *in vitro* cell experiments. Animal and cellular models have been used to explore mechanisms of oxidative damage in diabetes, such as streptozotocin, high-fat diets, and alloxan induced mice or rat, ob/ob mice, zucker diabetic fatty rat, as well as otsuka long-evans tokushima fatty rats (King, 2012). A review of polysaccharide applications for diabetes treatment for the past 5 years, revealed that the dose of polysaccharides ranged from 1 to 1,500 mg/kg/d in STZ or HFD induced diabetic models. Moreover, the polysaccharide and was mostly administered through intragastric administration. For example, the polysaccharide from *C. paliurus* was used as of 1, 10, and 100 mg/kg/d in STZ-induced mice (Zhang et al., 2021). After the administration of 1,500 mg/kg polysaccharide from *M. charantia* L (Zhang et al., 2019), diabetes symptoms improved. *In vivo* experiments of mice, polysaccharide doses were mainly distributed in the range of 100–800 mg/kg/d. How dosing is determined is unclear in some papers, but ultimately the results of these non-clinical trials seem to be very promising. We found that the dose of polysaccharides administered *in vitro* and *in vivo* seems to differ greatly, which seems to make it difficult to explain the specific effective dose of polysaccharides. In order to develop new treatments for diabetes with natural product characteristics that meet international standards, further clinical studies and evaluation of natural products, as well as structural and mechanism of action, as well as the relationship between biological outcomes and therapeutic effects, are needed. Using modern technology to develop more effective and economical methods to extract and separate polysaccharides, and expand the application of polysaccharides.

## Figures and Tables

**Table:** 

**ACC**	1-aminocyclopropane-1-carboxylic acid
**AGEs**	advanced glycosylation end products
**AKT**	Phosphorylated protein kinase B
**ALB**	albumin
**ALP**	alkaline phosphatase
**ALT**	alanine aminotransferase
**AST**	aspartate aminotransferase
**ATP**	adenosine triphosphate
**BAX**	Bcl-2-associated X protein
**BUN**	blood urea nitrogen
**CAT**	catalase
**CREA**	Creatinine
**eNOS**	endothelial nitric oxide synthase
**ERK**	extracellular signal-regulated kinase
**FOXO1**	forkhead box-1
**G6PC**	glucose-6-phosphatase
**GLUT**	glucose transporters
**GSH**	glutathione
**GSH-Px**	glutathione peroxidase
**GSK3β**	glycogen synthase kinase 3 beta
**H2O2**	hydrogen peroxide
**HDL**	High-density lipoprotein
**HO-1**	heme oxygenase-1
**INOS**	inducible nitric oxide synthase
**JNK**	Jun N-terminal kinases
**LDL**	low-density lipoprotein
**MDA**	malondialdehyde
**mTOR**	mechanistic target of rapamycin
**NF-кB**	nuclear transcription factor-kappa B
**NO**	nitric oxide
**Nox4**	NADPH oxidase 4
**NADPH**	quinone oxidoreductase 1
**Nrf2**	nuclear factor erythroid-2
**PEPCK**	phosphoenolpyruvate carboxylase
**PI3K**	phosphatidylinositol 3-k; inase
**PI3K**	phosphoinositide 3-kinase
**PKC**	protein kinase C
**PPARa**	Peroxisome proliferator-activated receptor alpha
**ROS**	reactive oxygen species
**SOD**	superoxide dismutase
**SREBP-1**	Sterol regulatory element binding protein-1
**TG**	triglyceride
**TC**	total cholesterol
**UA**	uric acid; Urea, carbamide
